# Pressure-support compared with pressure-controlled ventilation mitigates lung and brain injury in experimental acute ischemic stroke in rats

**DOI:** 10.1186/s40635-023-00580-w

**Published:** 2023-12-15

**Authors:** Adriana L. da Silva, Camila M. Bessa, Nazareth N. Rocha, Eduardo B. Carvalho, Raquel F. Magalhaes, Vera L. Capelozzi, Chiara Robba, Paolo Pelosi, Cynthia S. Samary, Patricia R. M. Rocco, Pedro L. Silva

**Affiliations:** 1 Laboratory of Pulmonary Investigation, Centro de Ciências da Saúde, Carlos Chagas Filho Institute of Biophysics, Federal University of Rio de Janeiro, Avenida Carlos Chagas Filho, S/N, Bloco G-014, Ilha Do Fundão, Rio de Janeiro, RJ 21941-902 Brazil; 2 Department of Physiology and Pharmacology, Biomedical Institute, Fluminense Federal University, Rio de Janeiro, Brazil; 3 Department of Pathology, Faculty of Medicine, University of São Paulo, São Paulo, Brazil; 4 Department of Surgical Sciences and Integrated Diagnostics (DISC), University of Genoa, Genoa, Italy; 5 Anesthesia and Critical Care, San Martino Policlinico Hospital, IRCCS for Oncology and Neurosciences, Genoa, Italy; 6 Department of Cardiorespiratory and Musculoskeletal Physiotherapy, Faculty of Physiotherapy, Federal University of Rio de Janeiro, Rio de Janeiro, Brazil

**Keywords:** Acute ischemic stroke, Mechanical ventilation, Lung injury, Brain injury

## Abstract

**Background:**

We aimed to evaluate the pulmonary and cerebral effects of low-tidal volume ventilation in pressure-support (PSV) and pressure-controlled (PCV) modes at two PEEP levels in acute ischemic stroke (AIS).

**Methods:**

In this randomized experimental study, AIS was induced by thermocoagulation in 30 healthy male Wistar rats. After 24 h, AIS animals were randomly assigned to PSV or PCV with V_T_ = 6 mL/kg and PEEP = 2 cmH_2_O (PSV-PEEP2 and PCV-PEEP2) or PEEP = 5 cmH_2_O (PSV-PEEP5 and PCV-PEEP5) for 2 h. Lung mechanics, arterial blood gases, and echocardiography were evaluated before and after the experiment. Lungs and brain tissue were removed for histologic and molecular biology analysis. The primary endpoint was diffuse alveolar damage (DAD) score; secondary endpoints included brain histology and brain and lung molecular biology markers.

**Results:**

In lungs, DAD was lower with PSV-PEEP5 than PCV-PEEP5 (*p* < 0.001); interleukin (IL)-1β was lower with PSV-PEEP2 than PCV-PEEP2 (*p* = 0.016) and PSV-PEEP5 than PCV-PEEP5 (*p* = 0.046); zonula occludens-1 (ZO-1) was lower in PCV-PEEP5 than PCV-PEEP2 (*p* = 0.042). In brain, necrosis, hemorrhage, neuropil edema, and CD45 + microglia were lower in PSV than PCV animals at PEEP = 2 cmH_2_O (*p* = 0.036, *p* = 0.025, *p* = 0.018, *p* = 0.011, respectively) and PEEP = 5 cmH_2_O (*p* = 0.003, *p* = 0.003, *p* = 0.007, *p* = 0.003, respectively); IL-1β was lower while ZO-1 was higher in PSV-PEEP2 than PCV-PEEP2 (*p* = 0.009, *p* = 0.007, respectively), suggesting blood–brain barrier integrity. Claudin-5 was higher in PSV-PEEP2 than PSV-PEEP5 (*p* = 0.036).

**Conclusion:**

In experimental AIS, PSV compared with PCV reduced lung and brain injury. Lung ZO-1 reduced in PCV with PEEP = 2 versus PEEP = 5 cmH_2_O, while brain claudin-5 increased in PSV with PEEP = 2 versus PEEP = 5 cmH_2_O.

**Supplementary Information:**

The online version contains supplementary material available at 10.1186/s40635-023-00580-w.

## Background

Patients with acute ischemic stroke (AIS) are frequently admitted to the intensive care unit [1–4]. Patients with AIS are at risk for pulmonary complications such as pneumonia and neurogenic edema, and may require respiratory support to protect the airways and optimize arterial blood gas exchange [1–3, 5]. However, there is a lack of consensus on how to set the ventilatory parameters under mechanical ventilation.

Pressure-controlled ventilation (PCV) and pressure-support ventilation (PSV) have been used worldwide in patients with neurologic disease [6]. During PCV, sedation is often increased, and inspiratory effort reduced, which may lead to hemodynamic impairment as well as lung and respiratory muscle dysfunction [7]. During PSV, less sedation is needed with better hemodynamics [8]. During assisted spontaneous ventilation, such as PSV, pleural pressure decreases, leading to tensile stress [9], whereas during PCV, a positive increase in pleural pressure is observed, resulting in compressive stress [10]. Pleural pressure during assisted spontaneous breathing effectively decreases intracardiac pressures [11], thus increasing venous return and contributing to heart–lung interaction. In addition, for the same tidal volume (V_T_), PSV reduces both atelectasis and heterogeneity when compared to PCV [12]. The changes in hemodynamics, whether by increasing blood flow or promoting venous congestion, may have positive or negative consequences on the brain and lungs, organs that are sensitive to blood flow. In addition, there is no consensus about the use of positive end-expiratory pressure (PEEP) in AIS. PEEP may not induce detrimental effects on intracranial pressure when set appropriately according to respiratory mechanics and lung imaging [13, 14].

The present study sought to evaluate the effects of mechanical ventilation with low V_T_ (6 mL/kg) under PSV and PCV at two different levels of PEEP (2 and 5 cmH_2_O) on lung and brain damage in experimental AIS. We hypothesized that PSV, compared with PCV, might improve diffuse alveolar damage (DAD) rather than induce further brain injury, regardless of the PEEP level.

## Methods

### Study approval

This prospective, randomized experimental study was approved by the Institutional Ethical Animal Care and Use Committee (CEUA CCS-017/19) of the Federal University of Rio de Janeiro (UFRJ) Health Sciences Center, Rio de Janeiro, Brazil. The principles of laboratory animal care proposed by the National Society for Medical Research (now the National Association for Biomedical Research) and the U.S. National Academy of Sciences *Guide for the Care and Use of Laboratory Animals* were followed throughout. Reporting followed the ARRIVE guidelines [15].

### Animal preparation

Animals were maintained at a fixed temperature (23 °C) and 12–12 h light–dark cycle. Free access to water and food was provided. After appropriate acclimation, 30 healthy Wistar rats (all male; weight 370 ± 12 g) were anesthetized by intraperitoneal (i.p.) injection of xylazine (2.5 mg/kg) and ketamine (75 mg/kg) and secured in a stereotactic frame. Ischemic stroke was then induced by thermocoagulation of pial blood vessels overlying the somatosensory, motor, and primary sensorimotor cortices, as previously described [16, 17]. In order to guarantee the same stroke severity pattern, the procedure was always performed by the same investigator (A.L.S.).

### Experimental protocol

Twenty-four hours after stroke induction, midazolam (1–2 mg/kg, i.p.) and ketamine (100 mg/kg, i.p.) were administered. A 24G catheter (Jelco^®^, BD, Franklin Lakes, NJ, USA) was placed in the tail vein, and total intravenous anesthesia was induced and maintained with midazolam (2 mg/kg/h) and ketamine (50 mg/kg/h). A continuous infusion of Ringer’s lactate (10 ml/kg/h; B. Braun, Crissier, Switzerland) was maintained throughout the experiment [18, 19]. Experiments were initiated once motor responses to stimuli, such as noise (handclap), whisker stimulation, and tail clamping, were absent. Animals were breathing spontaneously with no respiratory effort or gasping. The plane of anesthesia was monitored by MAP, heart rate, and RR during the experiment.

Animal temperature was kept at 37.5 °C ± 1 °C with a heating bed (EFF 421, Insight, Ribeirão Preto, Brazil). After local anesthesia (lidocaine 1.0%), tracheostomy was performed and a polyethylene cannula was introduced into the trachea. The right internal carotid artery was catheterized (18G; Arrow International, Reading, PA, USA) for blood gas analysis and MAP monitoring (Networked Multiparameter Veterinary Monitor LifeWindow 6000 V; Digicare Animal Health, Boynton Beach, FL, USA). Animals were attached to an airway pressure transducer (UT-PDP-70; SCIREQ, Montreal, Canada) and a two-sidearm pneumotachograph [20] was connected to a differential pressure transducer (UT-PDP-02; SCIREQ), for measurement of the airflow (V′).

Once the animals were hemodynamically stable (MAP > 100 mmHg), mechanical ventilation was started (Servo-i; Getinge AB, Göteborg, Sweden) via PSV for 5 min. During this 5-min period, delta pressure was constantly adjusted to reach a V_T_ = 6 mL/kg, end-expiratory pressure of zero, and fraction of inspired oxygen (FiO2) of 0.25 for all animals. Thus, all animals had the same starting point prior to randomization into different groups. Animal preparation was performed by an experienced researcher (A.L.S.), which also contributed to similar preparatory times.

**Fig. 1:**
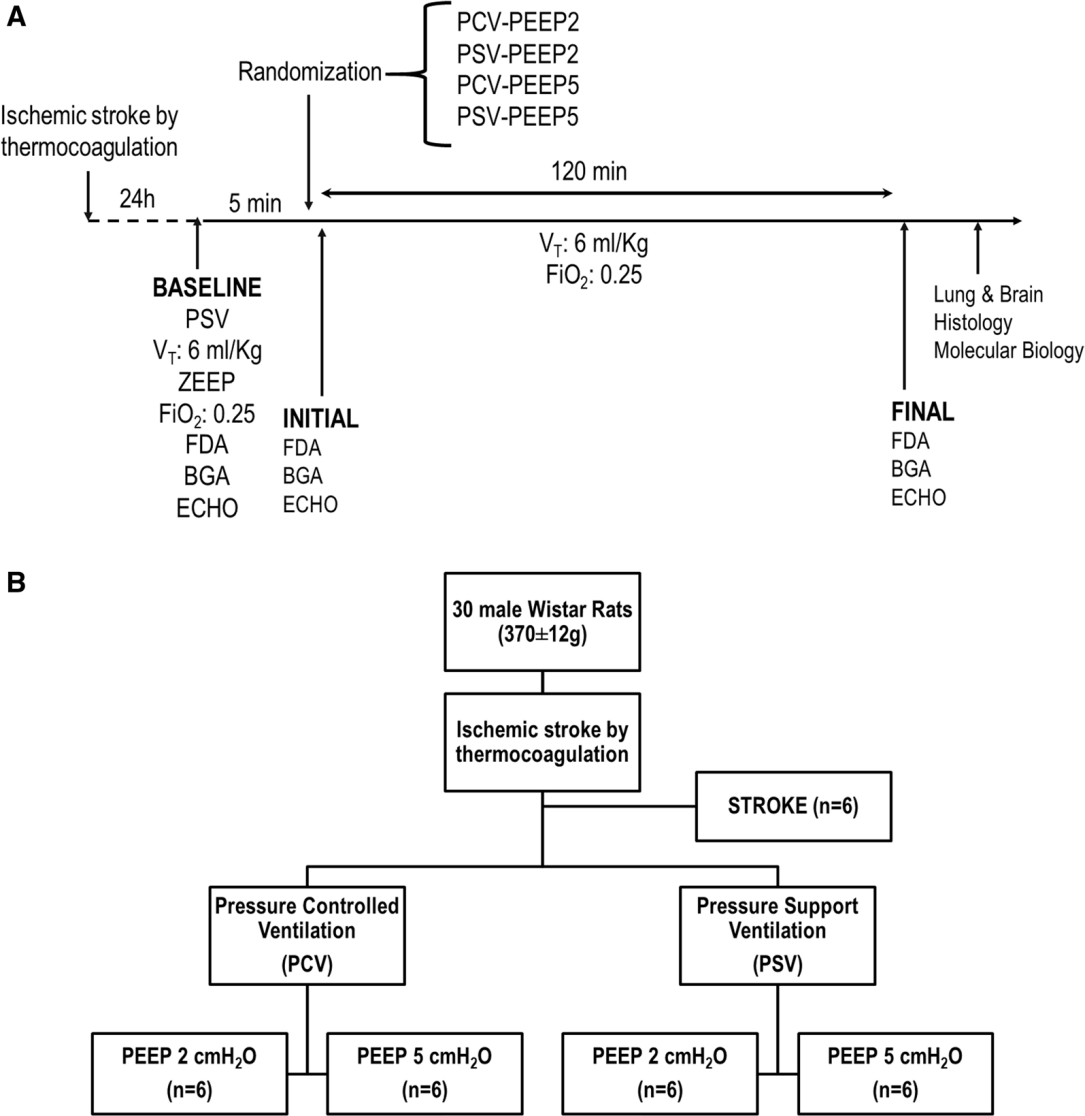
**A** Timeline representation of the experimental protocol. Respiratory system mechanics, arterial blood gases, and echocardiography were evaluated at the INITIAL and FINAL time points. **B** Schematic flowchart of the study design. BGA, blood gas analysis; ECHO, echocardiography; FDA, functional data acquisition; FiO_2_, fraction of inspired oxygen; PCV, pressure-controlled ventilation; PEEP, positive end-expiratory pressure; PSV, pressure-support ventilation; V_T_, tidal volume

At BASELINE, arterial blood (300 μL) was drawn for blood gas analysis. MAP, body temperature, and respiratory parameters were also gathered. Transthoracic echocardiography was performed (Fig. 1A). Five minutes after BASELINE, the animals were randomly assigned by the sealed-envelope method to the following ventilatory strategies (*n* = 6 animals/ventilatory strategy): PCV under PEEP of 2 cmH_2_O (PCV-PEEP2); PCV under PEEP of 5 cmH_2_O (PCV-PEEP5); PSV under PEEP of 2 cmH_2_O (PSV-PEEP2); and PSV under PEEP of 5 cmH_2_O (PSV-PEEP5). During PCV, pancuronium bromide (2 mg/kg; Cristália, Itapira, SP, Brazil) was intravenously administered. V_T_ was maintained aiming 6 mL/kg by constantly adjusting the delta pressure as mentioned earlier, for the PSV and PCV strategies. Six animals subjected to ischemic stroke but not ventilated (STROKE) were used for molecular biology analysis (Fig. 1B).

Data on blood gas analysis, functional data analysis, and echocardiography were collected 2 min after randomization, at INITIAL, and after 2 h of mechanical ventilation (FINAL). At FINAL, heparin (1,000 IU) was then administered into the tail vein, followed 2 min later by i.v. injection of sodium thiopental (100 mg/kg, Cristália), after which a laparotomy was performed to access the aorta for terminal exsanguination. The full process lasted around 5 min; the procedure and duration were the same in all animals. The brain and lungs were removed (the latter at an airway pressure equivalent to PEEP) for histologic and molecular biology analyses.

### Respiratory data acquisition and processing

Airflow and airway pressure were recorded continuously by running software written in LabVIEW (National Instruments; Austin, TX, USA). All signals were filtered (200 Hz), amplified by a 4-channel conditioner (SC-24, SCIREQ), and sampled at 200 Hz with a 12-bit analog-to-digital converter (National Instruments). V_T_ was obtained by integration of inspiratory airflow. The RR was estimated from Pes swings. Airway peak pressure (Ppeak,_RS_), plateau pressure (Pplat,_RS_), and driving pressure (∆P,_RS_) were obtained after 3 s of inspiratory pause, when the airflow was zero [12]. P_0.1_ was computed and represented the esophageal pressure obtained 100 ms after initiation of inspiratory effort. The respiratory data were assessed by two researchers (A.L.S. and C.M.B.) blinded to group allocation by a routine written in MATLAB (version R2007a; MathWorks Inc, Natick, MA, USA).

### Echocardiography and left carotid Doppler

This assessment was performed by an expert (N.N.R.) blinded to group allocation, using a 7.5-MHz probe (UGEO HM70A, 8–13 MHz transducer; Samsung). The following parameters were analyzed by transthoracic echocardiography: heart rate, right ventricular systolic volume, left ventricular systolic volume, cardiac output, and left carotid peak systolic velocity. Measurements were obtained from transthoracic parasternal and short-axis views, as recommended [21, 22].

### Histology

#### Lung

The left lung was fixed in 4% buffered formalin and paraffin-embedded for microtomy into 3 μm-thick sections, which were stained with hematoxylin and eosin. To obtain a panoramic view of the lung parenchyma, they were completely scanned (3DHISTECH, Budapest, Hungary). Photomicrographs at × 25, × 100, and × 400 magnification were obtained from 10 non-overlapping fields of view per section in the digitalized images. DAD was quantified by one expert pathologist (V.L.C.) who was blinded to group allocation. Six features—atelectasis, overdistension, interstitial edema, hemorrhage, thrombosis, and inflammation—were graded on scales of 0 to 4 in terms of severity (0, no effect; 4, maximum severity) and extent (0, not visualized; 4, (complete involvement). Final scores, ranging from 0 to 16, were then calculated as the product of the severity and extent of each feature. The cumulative DAD score thus ranged from 0 to 96 [16, 23].

#### Brain

Brains were fixed in 4% paraformaldehyde for 24 h and paraffin-embedded for microtomy into section 3 μm thick. The histoarchitecture of the hypothalamus was evaluated by hematoxylin and eosin staining, followed by immunostaining for CD11b + and CD45 + cells. CD11b + cells comprised mononuclear phagocytes, which were divided further into two populations: CD11b + cells characterized by microglia that reside in the dentate gyrus of the hippocampus parenchyma, and CD45 + cells that reside in the choroid plexus (internal pyramidal cells). These represented mainly myeloid-derived macrophages that reside at the interfaces of the brain and periphery [24].

Quantification was done using a weighted scoring system, adapted from a previous study [25], to represent the severity of brain tissue injury (necrosis, hemorrhage, neuropil edema, CD11b + dentate gyrus, CD11b + pyramidal, and CD45 + microglia), ranging from 0 (no injury) to 4 (severe injury). The extension of each feature was ranked from 0 (not visualized) to 4 (complete involvement). Again, sum scores ranging from 0 to 16 were calculated as the product of the severity and extent of each feature. The quantification was done by one expert pathologist (V.L.C.) who was blinded to group assignment.

### Biological markers in lung and brain tissues

Reverse transcriptase-polymerase chain reaction was used to measure biological markers in the lung and perilesional brain tissue [26]. In the lungs, markers associated with inflammation (interleukin [IL]-1β), epithelial cell damage (ZO-1), and (surfactant protein [SP]-B) were evaluated. In the brain, markers associated with inflammation (IL-1β), and blood–brain barrier integrity (ZO-1, claudin-5) [27] were evaluated. Additional file 1: Table S1 lists the primer sequences. Total RNA was extracted from frozen lung and brain sections tissues with the ReliaPrep™ RNA Tissue Miniprep System (Promega Corporation, Fitchburg, WI, USA), following manufacturer recommendations. RNA concentration measurement, cDNA synthesis, and relative mRNA quantitation were performed as described elsewhere [23]. Samples were measured in triplicate. For each sample, the expression of each gene was normalized to the *36B4* housekeeping gene and expressed as the fold change relative to STROKE, using the 2^−ΔΔCt^ method, where ΔCt = Ct (target gene) − Ct (reference gene) [28].

### Statistical analysis

Six animals per group would give adequate power (1 − β = 0.8) to identify significant differences (*p* < 0.05) in the DAD score (the primary endpoint) between SHAM and STROKE, according to a previous study [23], taking into account an effect size *d* = 2.0, a two-sided test, and a sample size ratio = 1 (G ∗ Power 3.1.9.2; University of Düsseldorf, Düsseldorf, Germany).

Normality was verified by the Kolmogorov–Smirnov test with Lilliefors’ correction, and homogeneity of variances, by the Levene median test. The primary endpoint was the DAD score, and secondary endpoints consisted of blood gas exchange, respiratory variables, and brain biological markers, as well as brain histology. The respiratory variables and blood gas exchange data were analyzed by two-way ANOVA followed by the Holm–Šidák multiple comparisons test to compare parameters among the groups and over time (INITIAL and FINAL). One-way ANOVA followed by the Holm–Šidák multiple comparisons was done to compare parametric data. The Kruskal–Wallis test followed by Dunn’s multiple comparisons test was used to compare nonparametric data obtained at the end of the experiment. Spearman correlation was done between brain biological markers and cardiac output. A *p* value < 0.05 was considered significant.

## Results

All animals survived to the end of the experiment (FINAL) and there were no missing data. Mean arterial pressure (MAP) remained ≥ 70 mmHg (minimal value, 97 ± 38 mmHg; maximal value, 118 ± 26 mmHg) during the experiments (Additional file 1: Table S2). At FINAL, no differences were observed in cumulative fluids (Additional file 1: Table S2). At INITIAL, left carotid peak systolic velocity was lower in the both PCV and PSV groups at PEEP of 5 cmH_2_O compared with PEEP of 2 cmH_2_O (Additional file 1: Table S3). No differences were observed in oxygenation (PaO_2_/FiO_2_; minimal value, 340 ± 39 mmHg, maximal value, 479 ± 69 mmHg) (Additional file 1: Table S4).

### Lung

**Fig. 2:**
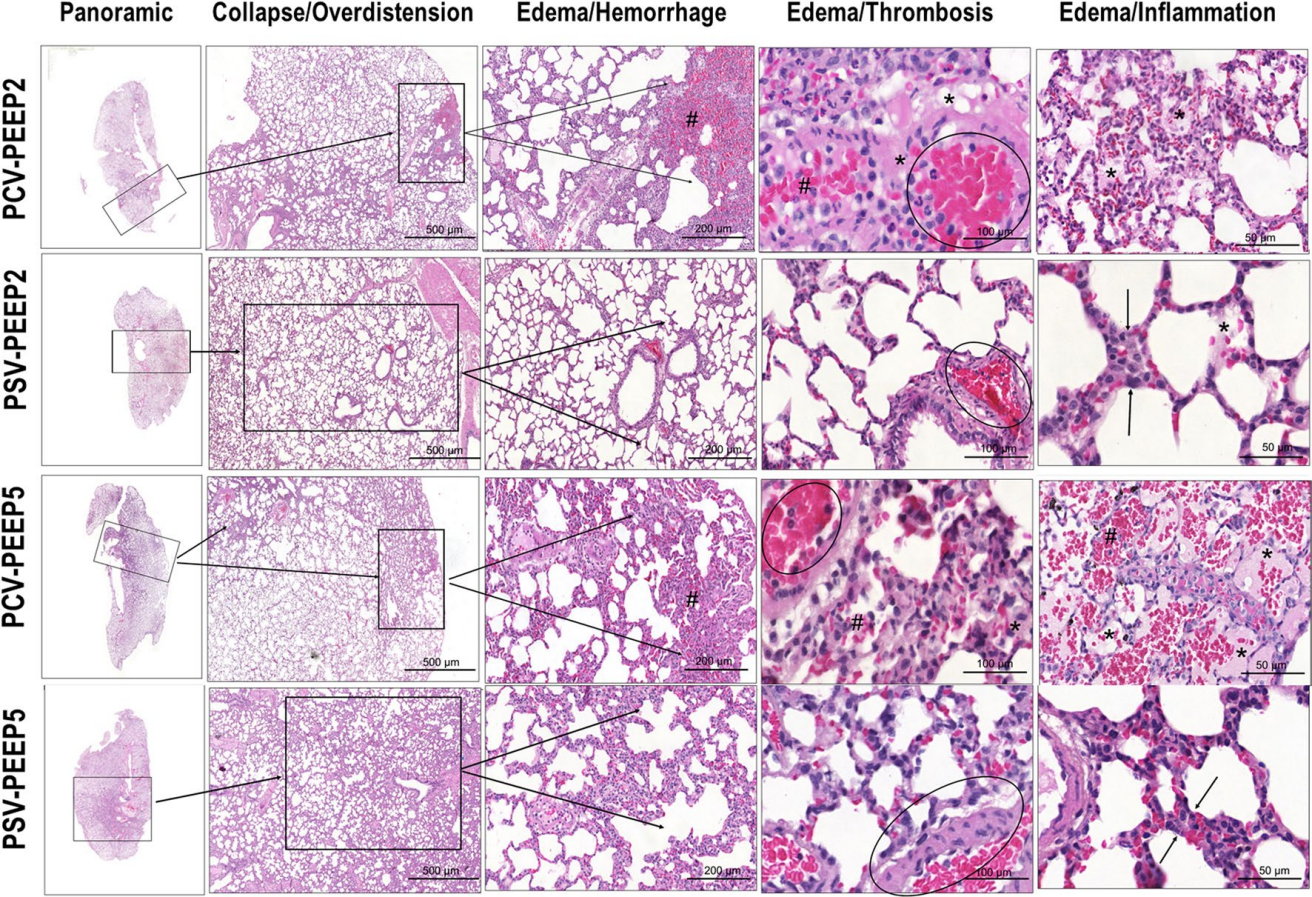
Histoarchitecture of the lung. Representative images stained with hematoxylin and eosin (HE) according to diffuse alveolar damage (DAD) features: collapse/overdistension; edema/hemorrhage; edema/thrombosis; edema/inflammation. PCV, pressure-controlled ventilation; PEEP, positive end-expiratory pressure; PSV, pressure-support ventilation

**Fig. 3:**
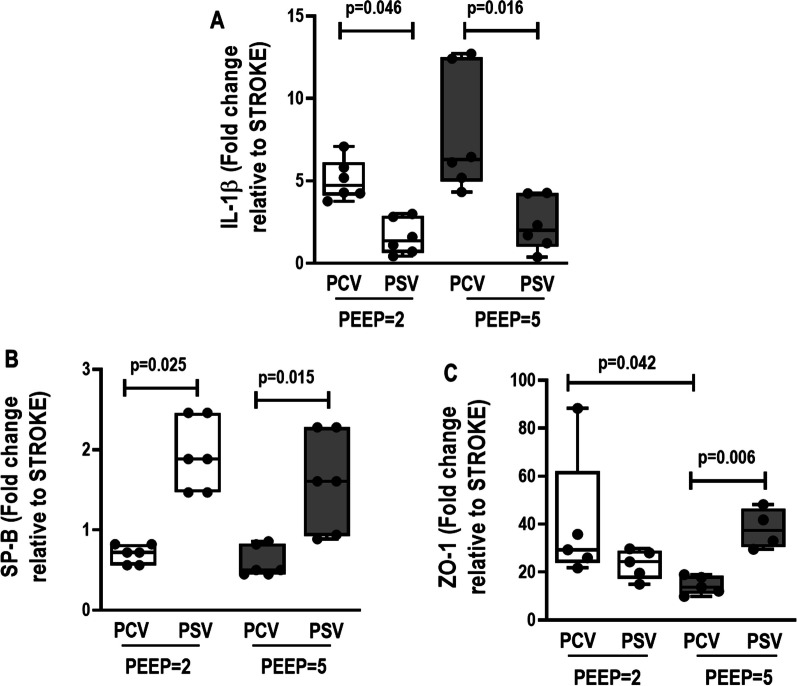
Expression of biological markers in the lung related to inflammation (interleukin [IL]-1β), epithelial cell damage (surfactant protein-B [SP-B]), and endothelial cell damage (zonula occludens-1 [ZO-1]). Boxes show the interquartile range (25% to 75%), whiskers encompass the range (minimum to maximum), and horizontal lines represent median values of 6 animals/group. Comparisons were done by Kruskal–Wallis test followed by Dunn’s multiple comparisons test (*p* < 0.05)

No differences were observed in V_T_ among the groups and over time. P_0.1_ did not differ between PSV groups (Additional file 1: Table S3). At FINAL, the respiratory rate (RR) was higher in PSV-PEEP2 compared with PCV-PEEP2 (92 ± 16 bpm *versus* 66 ± 11 bpm, *p* = 0.023). At INITIAL, Ppeak,_RS_ and Pplat,_RS_ were higher in both PCV-PEEP5 (16 ± 4, 13.6 ± 2.7 cmH_2_O, respectively) and PSV-PEEP5 (14.5 ± 3.3, 12.9 ± 2.3 cmH_2_O, respectively) compared with PCV-PEEP2 (11.3 ± 3.2, 9.9 ± 2.4 cmH_2_O, respectively) and PSV-PEEP2 (10.5 ± 1.8, 9.3 ± 1.6 cmH_2_O, respectively) (Additional file 1: Table S3). Interstitial edema and inflammation were lower in PSV-PEEP2 than PCV-PEEP2 (*p* = 0.029 and *p* = 0.045, respectively). Atelectasis, overdistension, interstitial edema, hemorrhage, thrombosis, and inflammation were lower in PSV-PEEP5 than PCV-PEEP5 (*p* = 0.002, *p* = 0.004, *p* = 0.011, *p* = 0.008, *p* = 0.004, and *p* = 0.030, respectively). The cumulative DAD score was lower in PSV-PEEP5 (median, 7; interquartile range [IQR], 5.75–8.25) than in PCV-PEEP5 (median, 40.5; IQR, 35.5–50.5, *p* < 0.001), and in PSV-PEEP2 (median, 9; IQR, 7–10.25) than PCV-PEEP2 (median, 34; IQR, 29.25–37.25, *p* < 0.001) (Fig. 2, Table 1 and Additional file 1: Table S5). IL-1β was lower in PSV-PEEP2 than PCV-PEEP2 (*p* = 0.046), and PSV-PEEP5 compared with PCV-PEEP5 (*p* = 0.016). Surfactant protein-B (SP-B) was higher in both PSV groups compared with the PCV groups, regardless of the PEEP levels. Zonula ocludens-1 (ZO-1) was lower in PCV-PEEP5 than PCV-PEEP2 (*p* = 0.042), but was higher in PSV-PEEP5 compared with PCV-PEEP5 (*p* = 0.006) (Fig. 3).

**Table 1:** Diffuse alveolar damage score

	PCV-PEEP2	PSV-PEEP2	PCV-PEEP5	PSV-PEEP5
Atelectasis (0–16)	3.5 (2.0–4.5)	1.0 (0.0–1.3)	7 (4–12)#	1.0 (0.0–1.0)
Overdistension (0–16)	4.0 (3.8–4.5)	0.5 (0.0–1.0)	8.5 (4–13)#	1.0 (0.0–1.0)
Interstitial edema (0–16)	8.5 (7.0–9.8)*	2.0 (1.8–2.5)	8.5 (4–13)#	2.0 (1.8–2.0)
Hemorrhage (0–16)	4.0 (3.0–5.3)	1.0 (1.0–2.5)	5 (3–6.75)#	1.0 (1.0–1.3)
Thrombosis (0–16)	8.5 (7.5– 9.8)	2.0 (2.0–2.5)	8.5 (5.5–12)#	1.5 (1.0–2.0)
Inflammation (0–16)	4.0 (3.5.– 6.8)*	2.0 (1.0–2.0)	3.5 (2.0–4.0)#	1.0 (1.0–1.3)
Cumulative DAD (0–96)	34.0 (29.3–37.3)*	9.0 (7.0–10.3)	40.5 (35.5–50.5)#	7.0 (5.8–8.3)

Cumulative DAD score representing injury from atelectasis, overdistension, interstitial edema, hemorrhage, thrombosis and inflammation. PCV, pressure-controlled ventilation; PEEP, positive end-expiratory pressure; PSV, pressure-support ventilation. Values are given as medians (interquartile ranges) of 6 animals in each group. Comparisons were done by Kruskal–Wallis test followed by Dunn’s multiple comparisons test (*p* < 0.05). *Versus the PSV-PEEP2 group; #versus the PSV-PEEP5 group

### Brain

**Fig. 4:**
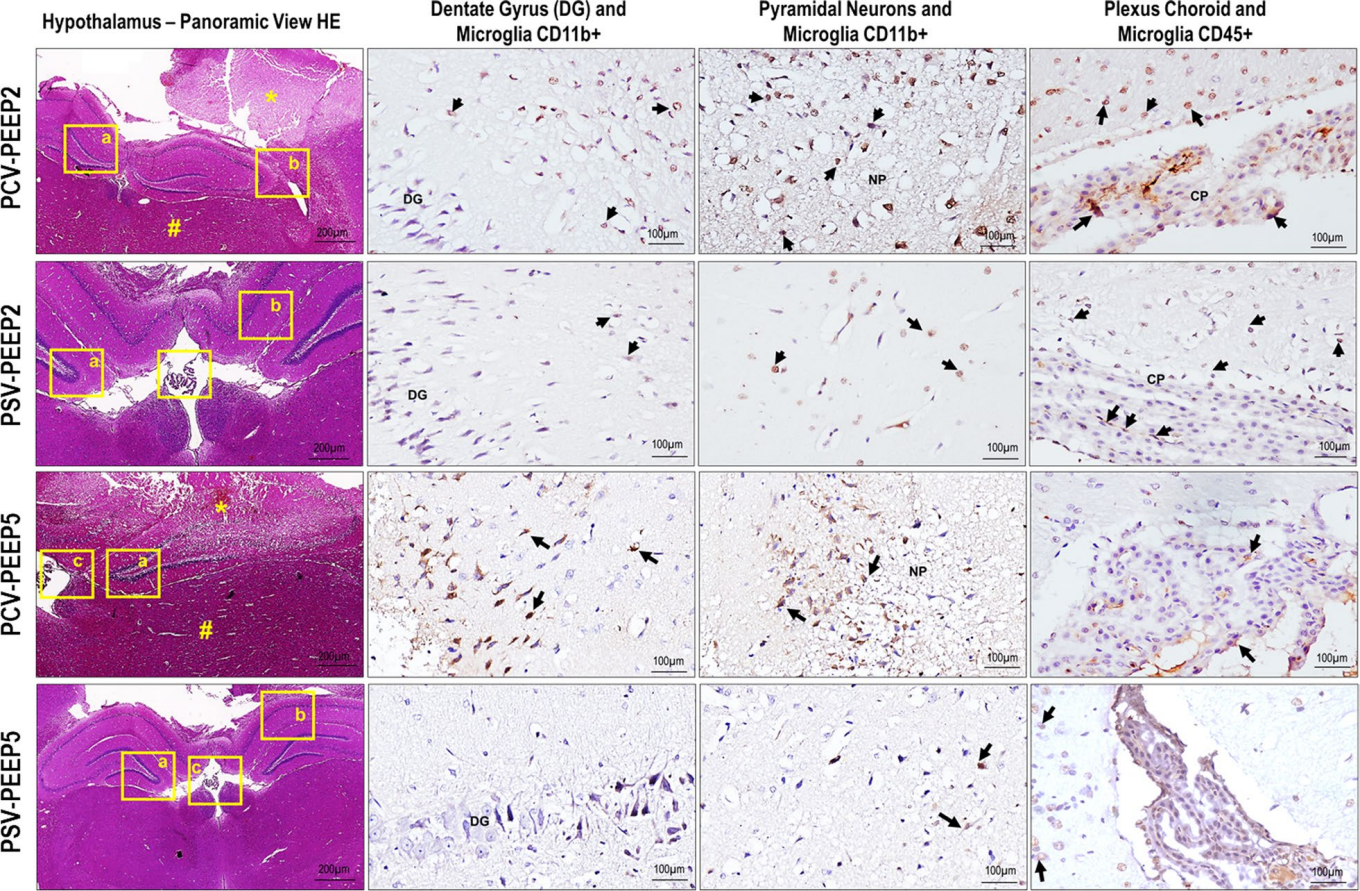
Histoarchitecture of the hypothalamus. Visualized at low magnification stained by hematoxylin and eosin and at high magnification immunostained for CD11b and CD45 in PCV-PEEP2, PSV-PEEP2, PCV-PEEP5, and PSV-PEEP5. PCV-PEEP2 and PCV-PEEP5 exhibiting necrosis (*) and hemorrhage (#) in the hippocampus distorting the curved histoarchitecture (**a,** lateral) of the pyramidal cells band encircling the dentate gyrus (**b,** lateral**)** and obstructing the choroid plexus. At high magnification, numerous CD11b + mononuclear phagocytes visualized in microglia (thin arrows) adjacent to the dentate gyrus (DG) of hippocampus parenchyma, and numerous CD45 + cells in the choroid plexus (CP, thick arrows), representing mainly myeloid-derived macrophages that reside at the interfaces of the brain and periphery. Compared with the PCV-PEEP5 group, the number of microglia and CD11b + myeloid cells, as well as choroid plexus CD45 +, was more abundant in the PCV-PEEP2 group in the three brain compartments of the hippocampus parenchyma including the DG, pyramidal neurons, and CP (arrows). Note the intense edema of the neuropils (NP) in the PCV group. PSV-PEEP2 and PSV-PEEP5 showing preserved hippocampus histoarchitecture as a multiply curved structure (**a,** middle, bottom) with pyramidal cells forming a band that circles the denser line of small cells comprising the DG (**b,** top**)** and CP (**c**, center). At high magnification, CD11b + cells comprised mononuclear phagocytes, which were divided further into two populations: CD11b + cells characterized by microglia (thin arrows) that reside in the DG of hippocampus parenchyma, and CD45 + cells that reside in the CP (thick arrows). They represented mainly myeloid-derived macrophages that reside at the interfaces of the brain and periphery. Compared with the PSV-PEEP5 group, the numbers of microglia and CD11b + myeloid cells, as well as Cp CD45 +, were more prominent in the PCV-P2 group in the three brain compartments of the hippocampus parenchyma, including the DG, pyramidal neurons and CP. PCV, pressure-controlled ventilation; PEEP, positive end-expiratory pressure; PSV, pressure-support ventilation

**Fig. 5:**
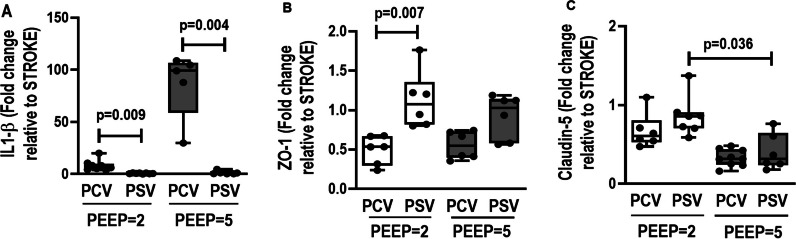
Expression of biological markers in the brain related to neuroinflammation (interleukin [IL]-1β), and endothelial cell damage (claudin-5 and zonula occludens-1 [ZO-1]) in the following groups: NV, nonventilated; PCV, pressure-controlled ventilation; PSV, pressure-support ventilation; PEEP, positive end-expiratory pressure. Relative gene expression was calculated as a ratio of the average gene expression levels compared with the reference gene (acidic ribosomal phosphoprotein P0 [36B4]) and expressed as fold change relative to nonventilated animals (NV). Boxes show the interquartile range (25–75%), whiskers encompass the range (minimum to maximum), and horizontal lines represent median values of 6 animals/group. Comparisons were done by Kruskal–Wallis test followed by Dunn’s multiple comparisons test (*p* < 0.05)

Necrosis, hemorrhage, neuropil edema, and CD45 + microglia were higher in PCV-PEEP2 compared with PSV-PEEP2 (*p* = 0.036, *p* = 0.025, *p* = 0.018, and *p* = 0.011, respectively). Necrosis, hemorrhage, neuropil edema, CD11b + dentate gyrus, CD11b + pyramidal, and CD45 + microglia were higher in PCV-PEEP5 compared with PSV-PEEP5 (*p* = 0.003, *p* = 0.003, *p* = 0.007, *p* = 0.005, *p* = 0.003, *p* = 0.001, respectively) (Fig. 4, Table 2 and Additional file 1: Table S6). IL-1β was higher in PCV-PEEP2 than PSV-PEEP2 (*p* = 0.009), and PCV-PEEP5 compared with PSV-PEEP5 (*p* = 0.004). ZO-1 gene expression was higher in PSV-PEEP2 than PCV-PEEP2 (*p* = 0.007). Claudin-5 was higher in PSV-PEEP2 than PSV-PEEP5 (*p* = 0.036) (Fig. 5).

**Table 2:** Histoarchitecture of the hypothalamus score

	PCV-PEEP2	PSV-PEEP2	PCV-PEEP5	PSV-PEEP5
Necrosis (0–16)	3.0 (2.0–4.0)*	1.0 (0.0–1.0)	6.0 (4.0–10.0)^**#**^	1.0 (0.0–1.0)
Hemorrhage (0–16)	4.0 (3.0–4.0)*	0.0 (0.0–1.0)	9.0 (6.0–14.0)^**#**^	1.0 (0.0–1.0)
Neuropil edema (0–16)	8.0 (6.0–9.0)*	2.0 (1.0–2.0)	9.0 (6.0–14.0)^**#**^	2.0 (1.0–2.0)
CD11b + dentate gyrus (0–16)	4.0 (3.0–4.0)	1.0 (1.0–4.0)	6.0 (3.5–7.5)^**#**^	1.0 (1.0–2.0)
CD11b + pyramidal (0–16)	8.0 (6.0– 9.0)*	2.0 (2.0–2.0)	8.0 (5.0–12.0)^**#**^	1.0 (1.0–2.0)
CD45 + microglia (0–16)	4.0 (4.0–8.0)*	2.0 (1.0–2.0)	3.0 (2.0–4.0)	1.0 (1.0–2.0)

Cumulative score representing hypothalamus injury from necrosis, hemorrhage, neuropil edema, CD11b + dentate gyrus, CD11b + pyramidal, and CD45 + microglia. PCV, pressure-controlled ventilation; PEEP, positive end-expiratory pressure; PSV, pressure-support ventilation. Values are given as medians (interquartile ranges) of 6 animals in each group. Comparisons were done using Kruskal–Wallis test followed by Dunn’s multiple comparisons test (*p* < 0.05). *Versus PSV-PEEP2 group; #versus PSV-PEEP5 group

**Fig. 6:**
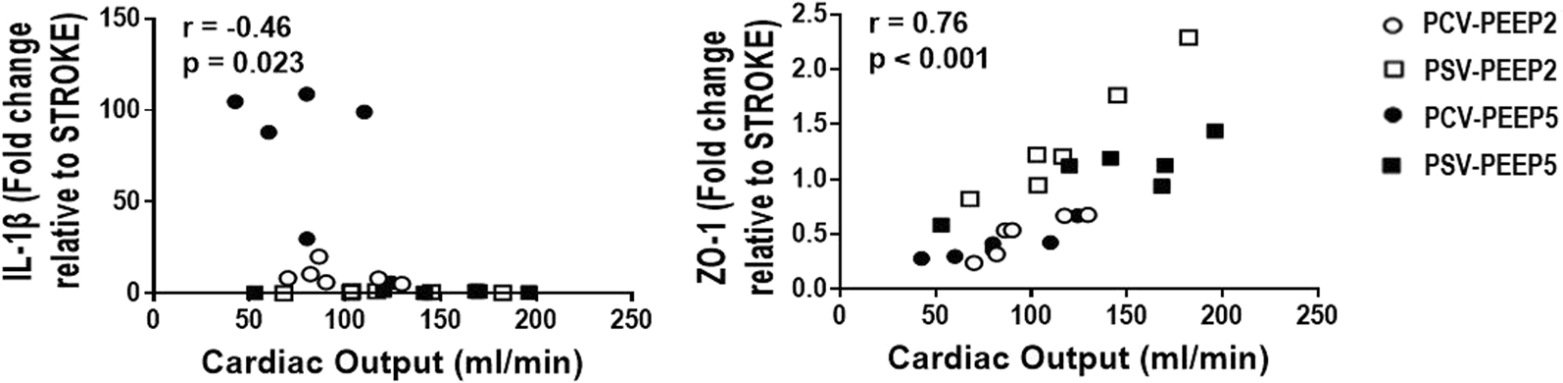
Cardiac output and brain damage correlation. Cardiac output was negatively correlated with interleukin (IL)-1β (*r* = − 0.46, *p* = 0.023) positively associated with Zonula occludens (ZO)-1 gene expression at brain tissue (*r* = 0.38, *p* = 0.068)

Cardiac output was negatively correlated with IL-1β (*r* = − 0.46, *p* = 0.023) and positively associated with ZO-1 gene expression (*r* = 0.76, *p* < 0.001) (Fig. 6).

## Discussion

In the present model of AIS, PSV compared with PCV (1) reduced the DAD score and markers of lung inflammation while increasing the gene expression of SP-B, regardless of the PEEP level; (2) decreased histologic features of brain necrosis, hemorrhage, and neuropil edema, regardless of the PEEP level; (3) reduced markers of brain inflammation regardless of the PEEP level; and (4) increased the expression of markers associated with blood–brain barrier protection (ZO-1) at a PEEP of 2 cmH_2_O. In addition, lung ZO-1 reduced in PCV with PEEP = 2 cmH_2_O compared with PEEP = 5 cmH_2_O, while brain claudin-5 increased in PSV with PEEP = 2 cmH_2_O compared with PEEP = 5 cmH_2_O.

In this study, we chose to use a model of focal AIS, instead of global ischemia, because this has a higher incidence in clinical practice, accounting for more than 90% of all strokes worldwide [29, 30]. In accordance with previous experimental studies [16, 23, 31], thermocoagulation of pial vessels over the primary sensorimotor cortices was chosen as the stroke model as it replicates the complex hemodynamic status and sensorimotor dysfunction 24 h after induction of AIS. Neurocritical patients usually require invasive mechanical ventilation after ischemic stroke [32] to ensure adequate airway protection and gas exchange [5]. Current knowledge suggests maintaining a protective V_T_ of 6–8 mL/kg of predicted body weight in patients with AIS. Hemodynamics may differ according to mechanical ventilation strategies and the level of sedation. PCV associated with high levels of sedation impairs hemodynamics and may lead to respiratory muscle dysfunction [7]. During PSV, hemodynamics are preserved due to less sedation [8], which may protect cerebral blood flow without affecting pH, PaCO_2_, and oxygenation [33]. Low or high PEEP levels may also have detrimental effects on cardiorespiratory function. In the present study, PEEP levels of 2 and 5 cmH_2_O were evaluated; human levels would require a 2-to-threefold increase, i.e., 6 and 15 cmH_2_O [34]. A prospective study on patients with traumatic brain injury demonstrated that increasing PEEP up to 15 cmH_2_O can improve brain tissue oxygenation [35]. Similar levels have been observed in clinical surveys on patients with severe traumatic brain injury without intracranial hypertension [36]. Specific areas of the brain were analyzed, such as the dentate gyrus in the hippocampus and the choroid plexus. The dentate gyrus plays a critical role in learning and memory [37], while the choroid plexus produces cerebrospinal fluid via the ependymal cells and serves as a barrier in the brain separating blood from cerebrospinal fluid [38].

In the present study, the cumulative DAD score was lower with PSV than PCV, independent of the PEEP levels. At INITIAL, both PSV and PCV under PEEP of 5 cmH_2_O showed high Ppeak,_RS_ and Pplat,_RS_ compared the respective groups at PEEP of 2 cmH_2_O. Throughout the experiment, there was an overall time effect toward a reduction in Ppeak,_RS_ and Pplat,_RS_ and ∆P,_RS_, which prevented differences among the groups at FINAL. The improvement in respiratory system mechanics over time might reflect some recruitment effect. An increased DAD score is associated with increased alveolar heterogeneity and viscoelastic mechanical properties of the respiratory system [39]. This is in line with the adoption of protective mechanical ventilation in lungs that are prone to injury due to crosstalk between the brain and distal organs [40]. Even under protective mechanical ventilation (V_T_ = 6 mL/kg), the role of compressive stress compared with tensile stress should be considered as well as hemodynamics. During assisted spontaneous ventilation, such as PSV, pleural pressure decreases, leading to tensile stress [9], whereas during PCV, a positive increase in pleural pressure is observed, resulting in compressive stress [10]. In PSV, cardiac output was higher than in PCV, and this can be partially explained by the pleural pressure (negative in PSV). In PSV, pleural pressure is negative during the expiratory phase and even more negative during inspiration [41]. Hence, venous return is favored toward the thorax during physiologic inspiration. In PSV groups, cardiac output values were between 108 and 149 mL/min, in agreement with studies on rats under spontaneous breathing [42]. On the other hand, during PCV, even at a similar protective V_T_ value (6 mL/kg), pleural pressure is positive, thus reducing venous return and cardiac output. In PCV groups, cardiac output values were between 76 and 126 mL/min. Reduced cardiac output values are in line with pre-acinar capillary blood stasis and may increase the hydrostatic pressure in lung capillaries. Hydrostatic pressure induces a dysfunction of the pulmonary capillary endothelium, which is mediated by active second messenger responses and characterized by an imbalanced release of biological markers [43]. It has been shown that patients with hydrostatic edema fluid showed increased neutrophils and inflammatory cytokines in the bronchoalveolar lavage fluid due to various causes [44–46]. We observed an increase in IL-1β and a decrease in SP-B during PCV, which is in line with early dysfunction of endothelial and epithelial cells [47], respectively. This can further explain the greater reduction in gene expression of ZO-1 in PCV than PSV. Reduced levels of ZO-1 are consistent with interstitial and alveolar edema due to diminished epithelial and endothelial integrity [48].

In the brain, we observed an increase in necrosis, hemorrhage, and neuropil edema in the hypothalamus in the PCV and PSV groups with similar PEEP levels. Positive pleural pressure, as seen in PCV, may lead to significant changes in systemic venous congestion (termed “backward failure”), which raises jugular venous pressure; this, in turn, is transmitted to the cerebral circulation [49]. It has been shown that cerebral venous congestion promotes blood–brain barrier disruption and neuroinflammation [50]. We observed an increase in IL-1β as well as a decrease in ZO-1 gene expression in the PCV compared with the PSV groups. Furthermore, the PCV groups showed reduced cardiac output levels (76–126 mL/min), which may be associated with increased CD11b + cells in the dentate gyrus and pyramidal hippocampus. CD11b + is a marker of activated microglia, representing the key immune effector cells of the central nervous system. Microglia cells can be activated by alterations in brain homeostasis leading to morphological and molecular changes. We may infer that the alteration in systemic venous congestion due to changes in pleural pressure and heart–lung interaction during PCV is likely able to change microglia activation. We also observed an increase in CD45 + in microglia cells, which is in line with the M1 activation phenotype, which releases inflammatory mediators and induces inflammation and neurotoxicity [51]. This is in line with our molecular biology of the brain tissue. Apart from hemodynamics, vagus nerve activation may have played a role in the PSV group, although this could not be explored fully in our experimental setting. Injurious mechanical ventilation has been associated with transient receptor potential cation channel subfamily V member 4 receptors (mechanoreceptors) activation. Following that, the pulmonary afferent purinergic receptors (vagal afferent receptors) may be activated, which may increase dopamine release in the hippocampus and triggering the intrinsic apoptotic cascade [52, 53]. Thus, it is expected that, during PSV, vagus nerve activity may be more physiological, while during PCV, it would tend toward the non-physiological. The level of sedation and anesthesia may also play a role.

### Limitations

This study has some limitations that need to be mentioned. First, the depth of sedation and anesthesia may have affected the neurological outcomes presented by PCV group in comparison to PSV group (Additional file 1: Table S7). Second, the study design precluded neurofunctional status evaluation. However, markers of inflammation and endothelial cell injury within perilesional brain tissue as well as morphological features and markers of microglia activation were analyzed. Third, we evaluated experimental AIS by thermocoagulation, therefore the results cannot be extrapolated to other models. Fourth, the rats were previously healthy, young, and male, and the data may not be directly extrapolated to the complexity of clinical practice. Fifth, we did not evaluate cerebral blood flow velocity; instead, we measured cardiac output and left carotid peak systolic velocity as surrogates. Sixth, no differences in ventilator-induced lung injury susceptibility have been associated with sex [54], a finding that supports the inclusion of both sexes [55] instead of justifying the use of single-sex samples in experimental studies. Whether sex would have an impact on brain damage after ventilation in PSV or PCV should be studied in future preclinical research.

## Conclusions

In experimental AIS, PSV compared with PCV reduced lung and brain injury. In addition, lung ZO-1 was reduced in PCV with PEEP = 2 cmH_2_O versus PEEP = 5 cmH_2_O, while brain claudin-5 increased in PSV with PEEP = 2 cmH_2_O versus PEEP = 5 cmH_2_O.

### Supplementary Information

**Additional file 1.** Supplemental Material.

## Data Availability

The datasets used and/or analyzed during the current study are available from the corresponding author on reasonable request.

## Abbreviations

PEEP: Positive end-expiratory pressure
AIS: Acute ischemic stroke
PSV: Pressure-support ventilation
PCV: Pressure-controlled ventilation
DAD: Diffuse alveolar damage
IL: Interleukin
V_T_: Tidal volume
MAP: Mean arterial pressure
PaO_2_/FiO_2_: Ratio of arterial oxygen partial pressure to fractional inspired oxygen
Ppeak,RS: Peak airway pressure
Pplat_,RS_: Plateau pressure
ΔP_,RS_: Driving pressure
ZO: Zonula ocludens
SP-B: Surfactant protein-B
PaCO_2_: Arterial carbon dioxide partial pressure
CO: Cardiac output
ip: Intraperitoneal
iv: Intravenous
