# Supplementary material for: Pressure-support compared with pressure-controlled ventilation mitigates lung and brain injury in experimental acute ischemic stroke in rats
Source: Intensive Care Med Exp. 2023 Dec 15;11:93. doi: 10.1186/s40635-023-00580-w (PMC10724101; doi:10.1186/s40635-023-00580-w)
Supplement: Supplementary file 1 — Additional file 1. Supplemental Material. [file 40635_2023_580_MOESM1_ESM.docx]

Supplemental Material

Pressure-support compared with pressure-controlled ventilation mitigates lung and brain injury in experimental acute ischemic stroke in rats.

Adriana L. da Silva^1^, Camila M. Bessa^1^, Nazareth N. Rocha^1,2^, Eduardo B. Carvalho^1^, Raquel F. Magalhaes^1^, Vera L. Capelozzi^3^, Chiara Robba^4,5^, Paolo Pelosi^4,5^, Cynthia S. Samary^1,6^, Patricia R.M. Rocco^1^, Pedro L. Silva^1*^

^1^Laboratory of Pulmonary Investigation, Carlos Chagas Filho Institute of Biophysics, Federal University of Rio de Janeiro, Rio de Janeiro, Brazil

^2^Department of Physiology and Pharmacology, Biomedical Institute, Fluminense Federal University, Rio de Janeiro, Brazil

^3^Department of Pathology, Faculty of Medicine, University of São Paulo, São Paulo, Brazil

^4^Department of Surgical Sciences and Integrated Diagnostics (DISC), University of Genoa, Genoa, Italy

^5^Anesthesia and Critical Care, San Martino Policlinico Hospital, IRCCS for Oncology and Neurosciences, Genoa, Italy

^6^Department of Cardiorespiratory and Musculoskeletal Physiotherapy, Faculty of Physiotherapy, Federal University of Rio de Janeiro, Rio de Janeiro, Brazil

**SUPPLEMENTAL TABLE S1** Forward and reverse oligonucleotide sequences of target gene primers

| Gene | Primer | Sequence (5′–3′) |
| --- | --- | --- |
| Claudin-5 | Forward | CTC CGC AAG AGA CTT CCA G |
|  | Reverse | CTC CTC TCC GGA CTT GTG A |
| IL-1β | Forward | GAA GAA AAC AAAT GGA TCA AGG GAT T |
|  | Reverse | GGA GTG GAA ATG GCT CTA ATG AAC T |
| SP-B | Forward | TTG GGA CAG GGA AGA ACG A |
|  | Reverse | TCA ACG CTG GGC AAC ATT A |
| ZO-1 | Forward | CAC CAC AGA CAT CCA ACC AG |
|  | Reverse | CAC CAA CCA CTC TCC CTT GT |
| Housekeeping gene *36B4* | Forward | AAT CCT GAG CGA TGT GCA G |
|  | Reverse | GCT GCC ATT GTC AAA CAC |

IL-1β, interleukin-1 beta; SP-B, surfactant protein B; ZO-1, zonula occludens; *36B4*, acidic ribosomal phosphoprotein P0.

**SUPPLEMENTAL TABLE 2** Echocardiography and functional data acquisition at INITIAL and FINAL

|  | **PCV or PSV** | **PEEP** | **INITIAL** | **FINAL** | **Time effect** | **Group effect** | **Interaction** |
| --- | --- | --- | --- | --- | --- | --- | --- |
| **HR (bpm)** | PCV | 2 | 399 ± 27 | 413 ± 37 | 0.677 | 0.039 | 0.115 |
|  | PCV | 5 | 382 ± 35 | 375 ± 57 |  |  |  |
|  | PSV | 2 | 438 ± 28 | 387 ± 18 |  |  |  |
|  | PSV | 5 | 405 ± 46 | 447 ± 37† |  |  |  |
| **MAP (mmHg)** | PCV | 2 | 138 ± 17 | 107 ± 13 | <0.001 | 0.787 | 0.189 |
|  | PCV | 5 | 133 ± 18 | 97 ± 38 |  |  |  |
|  | PSV | 2 | 123 ± 29 | 118 ± 26 |  |  |  |
|  | PSV | 5 | 144 ± 11 | 106 ± 8 |  |  |  |
| **RVSV (μL)** | PCV | 2 | 361 ± 80 | 280 ± 68 | 0.858 | 0.368 | <0.001 |
|  | PCV | 5 | 240 ± 70 | 242 ± 96 |  |  |  |
|  | PSV | 2 | 335 ± 140 | 307 ± 119 |  |  |  |
|  | PSV | 5 | 248 ± 83 | 348 ± 50 |  |  |  |
| **LVSV (μL)** | PCV | 2 | 320 ± 46 | 242 ± 48 | 0.873 | 0.462 | <0.001 |
|  | PCV | 5 | 213 ± 88 | 277 ± 88 |  |  |  |
|  | PSV | 2 | 344 ± 79 | 280 ± 76 |  |  |  |
|  | PSV | 5 | 248 ± 89 | 334 ± 90 |  |  |  |
| **CO (mL/min)** | PCV | 2 | 126 ± 16† | 118 ± 18† | 0.746 | 0.045 | <0.001 |
|  | PCV | 5 | 76 ± 21 | 78 ± 22 |  |  |  |
|  | PSV | 2 | 149 ± 66 | 110 ± 41 |  |  |  |
|  | PSV | 5 | 108 ± 36 | 141 ± 50 |  |  |  |
| **Left carotid peak systolic velocity (cm/s)** | PCV | 2 | 87 ± 15 | 85 ± 9 | 0.002 | 0.697 | 0.034 |
|  | PCV | 5 | 72 ± 9* | 90 ± 18 |  |  |  |
|  | PSV | 2 | 94 ± 11 | 91 ± 5 |  |  |  |
|  | PSV | 5 | 66 ± 4# | 85 ± 3 |  |  |  |
| **Cumulative fluids (mL)** | PCV | 2 | - | 8.7 ± 1.4 |  |  |  |
|  | PCV | 5 | - | 8.4 ± 1.5 |  |  |  |
|  | PSV | 2 | - | 8.3 ± 2.7 |  |  |  |
|  | PSV | 5 | - | 8.9 ± 3.0 |  |  |  |

PCV, pressure-controlled ventilation; PSV, pressure support ventilation; PEEP, positive end-expiratory pressure; HR, heart rate; MAP, mean arterial pressure; RVSV, right ventricle systolic volume; LVSV, left ventricle systolic volume; CO, cardiac output; PEEP2=2 cmH_2_O; PEEP5=5 cmH_2_O. Comparisons were done by two-way ANOVA followed by Holm–Šidák multiple comparisons tests (*p*<0.05). Data are presented as means ± standard deviation of 6 animals/group. **versus* PCV-PEEP2; †*versus* PCV-PEEP5; #*versus* PSV-PEEP2.

**SUPPLEMENTAL TABLE 3** Respiratory variables at INITIAL and FINAL

|  | **PCV or PSV** | **PEEP** | **INITIAL** | **FINAL** | **Time effect** | **Group effect** | **Interaction** |
| --- | --- | --- | --- | --- | --- | --- | --- |
| V_T_ (mL/kg) | PCV | 2 | 6.2 ± 0.6 | 6.1 ± 0.6 | 0.135 | 0.207 | 0.127 |
|  | PCV | 5 | 6.6 ± 0.5 | 6.1 ± 0.4 |  |  |  |
|  | PSV | 2 | 6.3 ± 0.4 | 5.9 ± 0.2 |  |  |  |
|  | PSV | 5 | 6.6 ± 0.9 | 6.9 ± 0.8 |  |  |  |
| RR (bpm) | PCV | 2 | 63 ± 9 | 66 ± 11 | 0.123 | 0.066 | 0.654 |
|  | PCV | 5 | 73 ± 16 | 74 ± 14 |  |  |  |
|  | PSV | 2 | 84 ± 24 | 92 ± 16* |  |  |  |
|  | PSV | 5 | 80 ± 10 | 81 ± 10 |  |  |  |
| Ppeak,_RS_ (cmH_2_O) | PCV | 2 | 11.3 ± 3.2 | 11.9 ± 2.5 | 0.003 | 0.011 | 0.023 |
|  | PCV | 5 | 16.0 ± 4.0* | 14.1 ± 2.6 |  |  |  |
|  | PSV | 2 | 10.5 ± 1.8 | 9.6 ± 1.7 |  |  |  |
|  | PSV | 5 | 14.5 ± 3.3# | 12.7 ± 1.9 |  |  |  |
| Pplat,_RS_ (cmH_2_O) | PCV | 2 | 9.9 ± 2.4 | 10.5 ± 1.8 | 0.018 | 0.005 | 0.045 |
|  | PCV | 5 | 13.6 ± 2.7* | 12.7 ± 2.0 |  |  |  |
|  | PSV | 2 | 9.3 ± 1.6 | 8.6 ± 1.6 |  |  |  |
|  | PSV | 5 | 12.9 ± 2.3# | 11.1 ± 1.1 |  |  |  |
| ΔP,_RS_ (cmH_2_O) | PCV | 2 | 8.2 ± 2.9 | 8.3 ± 1.5 | 0.009 | 0.288 | 0.116 |
|  | PCV | 5 | 9.3 ± 3.3 | 7.7 ± 1.7 |  |  |  |
|  | PSV | 2 | 7.0 ± 1.8 | 6.7 ± 1.4 |  |  |  |
|  | PSV | 5 | 7.7 ± 2.1 | 5.8 ± 1.1 |  |  |  |
| P_0.1_ (cmH_2_O) | PCV  PCV | 2  5 | -  - | -  - | 0.227 | 0.790 | 0.507 |
|  | PSV  PSV | 2  5 | -1.4 ± 0.4  -1.2 ± 0.2 | -1.5 ± 0.5  -1.5 ± 0.4 |  |  |  |

PCV, pressure-controlled ventilation; PSV, pressure-support ventilation; PEEP, positive end-expiratory pressure; V_T_, tidal volume; RR, respiratory rate; Ppeak,_RS_, airway peak pressure; Pplat,_RS_, airway plateau pressure; ΔP,_RS_, airway driving pressure; P_0.1_: esophageal pressure measured after 100ms the beginning of inspiratory effort. Comparisons were done by two-way ANOVA followed by Holm–Šidák multiple comparisons tests (*p*<0.05). Data are presented as means ± standard deviation of 6 animals/group. *Versus PCV-PEEP2; #versus PSV-PEEP2.

**SUPPLEMENTAL TABLE 4** Blood gas exchange at INITIAL and FINAL

|  | **PCV or PSV** | **PEEP** | **INITIAL** | **FINAL** | **Time effect** | **Group effect** | **Interaction** |
| --- | --- | --- | --- | --- | --- | --- | --- |
| pHa | PCV | 2 | 7.45 ± 0.03 | 7.35 ± 0.02 | 0.992 | 0.28 | 0.098 |
|  | PCV | 5 | 7.36 ± 0.01 | 7.46 ± 0.12 |  |  |  |
|  | PSV | 2 | 7.35 ± 0.01 | 7.37 ± 0.02 |  |  |  |
|  | PSV | 5 | 7.37 ± 0.03 | 7.36 ± 0.02 |  |  |  |
| PaO_2_/FiO_2_ | PCV | 2 | 453 ± 28 | 479 ± 69 | 0.598 | 0.45 | 0.464 |
|  | PCV | 5 | 488 ± 64 | 452 ± 8 |  |  |  |
|  | PSV | 2 | 429 ± 93 | 340 ± 39 |  |  |  |
|  | PSV | 5 | 392 ± 57 | 416 ± 67 |  |  |  |
| PaCO_2_ (mmHg) | PCV | 2 | 29 ± 10 | 32 ± 12 | 0.709 | 0.628 | 0.165 |
|  | PCV | 5 | 38 ± 5 | 29 ± 2 |  |  |  |
|  | PSV | 2 | 34 ± 5 | 39 ± 6 |  |  |  |
|  | PSV | 5 | 40 ± 7 | 38 ± 4 |  |  |  |
| HCO_3_^−^ (mEq/L) | PCV | 2 | 20 ± 7 | 19 ± 6 | 0.848 | 0.878 | 0.146 |
|  | PCV | 5 | 21 ± 3 | 22 ± 7 |  |  |  |
|  | PSV | 2 | 18 ± 2 | 22 ± 3 |  |  |  |
|  | PSV | 5 | 40 ± 7 | 20 ± 2 |  |  |  |

Blood gas exchange at INITIAL and FINAL time points. PCV, pressure-controlled ventilation; PSV, pressure-support ventilation; pHa, arterial pH; PaO_2_/FiO_2_, arterial oxygen partial pressure divided by the oxygen fraction; PaCO_2_, arterial carbon dioxide partial pressure; HCO_3_^−^, bicarbonate. Comparisons were done by two-way ANOVA followed by Holm–Šidák multiple comparisons tests (*p*<0.05). Data are presented as means ± standard deviation of 6 animals/group.

**SUPPLEMENTAL TABLE 5**  Detailed statistical report for Dunn’s multiple comparisons (DAD score)

|  | **Comparisons** | **p value** |
| --- | --- | --- |
| Atelectasis | PSV-PEEP2 vs PSV-PEEP5 | 0.999 |
|  | PSV-PEEP2 vs PCV-PEEP2 | 0.141 |
|  | PSV-PEEP5 vs PCV-PEEP5 | 0.002 |
|  | PCV-PEEP2 vs PCV-PEEP5 | 0.999 |
| Overdistension | PSV-PEEP2 vs PSV-PEEP5 | 0.999 |
|  | PSV-PEEP2 vs PCV-PEEP2 | 0.051 |
|  | PSV-PEEP5 vs PCV-PEEP5 | 0.004 |
|  | PCV-PEEP2 vs PCV-PEEP5 | 0.999 |
| Interstitial edema | PSV-PEEP2 vs PSV-PEEP5 | 0.999 |
|  | PSV-PEEP2 vs PCV-PEEP2 | 0.029 |
|  | PSV-PEEP5 vs PCV-PEEP5 | 0.011 |
|  | PCV-PEEP2 vs PCV-PEEP5 | 0.999 |
| Hemorrhage  Thrombosis  Inflammation  Cumulative DAD score | PSV-PEEP2 vs PSV-PEEP5 | 0.999 |
|  | PSV-PEEP2 vs PCV-PEEP2 | 0.108 |
|  | PSV-PEEP5 vs PCV-PEEP5 | 0.008 |
|  | PCV-PEEP2 vs PCV-PEEP5 | 0.999 |
|  | PSV-PEEP2 vs PSV-PEEP5 | 0.999 |
|  | PSV-PEEP2 vs PCV-PEEP2 | 0.061 |
|  | PSV-PEEP5 vs PCV-PEEP5 | 0.004 |
|  | PCV-PEEP2 vs PCV-PEEP5 | 0.999 |
|  | PSV-PEEP2 vs PSV-PEEP5 | 0.999 |
|  | PSV-PEEP2 vs PCV-PEEP2 | 0.045 |
|  | PSV-PEEP5 vs PCV-PEEP5 | 0.030 |
|  | PCV-PEEP2 vs PCV-PEEP5 | 0.999 |
|  | PSV-PEEP2 vs PSV-PEEP5 | 0.999 |
|  | PSV-PEEP2 vs PCV-PEEP2 | <0.001 |
|  | PSV-PEEP5 vs PCV-PEEP5 | <0.001 |
|  | PCV-PEEP2 vs PCV-PEEP5 | 0.999 |

**SUPPLEMENTAL TABLE 6**  Detailed statistical report for Dunn’s multiple comparisons (hypothalamus injury score)

|  | **Comparisons** | **p value** |
| --- | --- | --- |
| Necrosis | PSV-PEEP2 vs PSV-PEEP5 | 0.999 |
|  | PSV-PEEP2 vs PCV-PEEP2 | 0.036 |
|  | PSV-PEEP5 vs PCV-PEEP5 | 0.003 |
|  | PCV-PEEP2 vs PCV-PEEP5 | 0.999 |
| Hemorrhage | PSV-PEEP2 vs PSV-PEEP5 | 0.999 |
|  | PSV-PEEP2 vs PCV-PEEP2 | 0.025 |
|  | PSV-PEEP5 vs PCV-PEEP5 | 0.003 |
|  | PCV-PEEP2 vs PCV-PEEP5 | 0.999 |
| Neuropil edema | PSV-PEEP2 vs PSV-PEEP5 | 0.999 |
|  | PSV-PEEP2 vs PCV-PEEP2 | 0.018 |
|  | PSV-PEEP5 vs PCV-PEEP5 | 0.007 |
|  | PCV-PEEP2 vs PCV-PEEP5 | 0.999 |
| CD11b+ dentate gyrus  CD11b+ pyramidal  CD45+ microglia | PSV-PEEP2 vs PSV-PEEP5 | 0.999 |
|  | PSV-PEEP2 vs PCV-PEEP2 | 0.230 |
|  | PSV-PEEP5 vs PCV-PEEP5 | 0.005 |
|  | PCV-PEEP2 vs PCV-PEEP5 | 0.999 |
|  | PSV-PEEP2 vs PSV-PEEP5 | 0.999 |
|  | PSV-PEEP2 vs PCV-PEEP2 | 0.051 |
|  | PSV-PEEP5 vs PCV-PEEP5 | 0.003 |
|  | PCV-PEEP2 vs PCV-PEEP5 | 0.999 |
|  | PSV-PEEP2 vs PSV-PEEP5 | 0.999 |
|  | PSV-PEEP2 vs PCV-PEEP2 | 0.011 |
|  | PSV-PEEP5 vs PCV-PEEP5 | 0.001 |
|  | PCV-PEEP2 vs PCV-PEEP5 | 0.999 |

**SUPPLEMENTAL TABLE 7**  Cumulative sedative and neuromuscular blockage among groups

|  | **Groups** | **Median [25-75%]** |
| --- | --- | --- |
| Sedation (mg/kg) | PCV-PEEP2 | 3.0 [0.9-3.7] |
|  | PSV-PEEP2 | 1.7 [1.2-2.4] |
|  | PCV-PEEP5 | 3.3 [1.4-3.6] |
|  | PSV-PEEP5 | 1.3 [1.0-2.2] |
| Neuromuscular blockage (mg/kg) | PCV-PEEP2 | 0.4 [0.3-0.6] |
|  | PSV-PEEP2 | 0.0 [0.0-0.0] |
|  | PCV-PEEP5 | 0.4 [0.4-0.6] |
|  | PSV-PEEP5 | 0.0 [0.0-0.0] |

Comparisons were done by Kruskal–Wallis test followed by Dunn’s multiple comparisons test (*p*<0.05).
